# Perceptions and Challenges of Telehealth Obstetric Clinics Among Pregnant Women in Hong Kong: Cross-Sectional Questionnaire Study

**DOI:** 10.2196/46663

**Published:** 2023-09-19

**Authors:** Ka Wang Cheung, Tiffany Sin-Tung Au, Joan Kar On Wai, Mimi Tin-Yan Seto

**Affiliations:** 1 Department of Obstetrics & Gynaecology Queen Mary Hospital Hong Kong China (Hong Kong); 2 Department of Obstetrics & Gynaecology The University of Hong Kong Hong Kong China (Hong Kong)

**Keywords:** delivery, digital, portable electronic applications, smartphone, pregnancy, virtual clinics, telehealth, telemedicine

## Abstract

**Background:**

Integrating telehealth in an obstetric care model is important to prepare for possible infection outbreaks that require social distancing and limit in-person consultations. To ensure the successful implementation of obstetric telehealth in Hong Kong, it is essential to understand and address pregnant women’s concerns.

**Objective:**

This study aimed to assess pregnant women’s attitudes, concerns, and perceptions regarding telehealth obstetric clinic services in Hong Kong.

**Methods:**

We conducted a prospective cross-sectional questionnaire study at Queen Mary Hospital between November 2021 and August 2022. Utilizing a 5-point rating scale, the questionnaire aimed to capture pregnant women’s preferences, expectations, feasibility perceptions, and privacy concerns related to telehealth clinic services. We used statistical analyses, including chi-square tests and multinomial logistic regression, to compare questionnaire responses and investigate the association between advancing gestation and attitudes toward telehealth clinics.

**Results:**

The study included 664 participants distributed across different pregnancy stages: 269 (40.5%) before 18 gestational weeks, 198 (29.8%) between 24 and 31 weeks, and 197 (29.7%) after delivery. Among them, 49.8% (329/664) favored face-to-face consultations over telehealth clinics, and only 7.3% (48/664) believed the opposite. Additionally, 24.2% (161/664) agreed that telehealth clinics should be launched for obstetric services. However, the overall preference for telehealth clinics was <20% for routine prenatal checkups (81/664, 12.2%) and addressing pregnancy-related concerns, such as vaginal bleeding (76/664, 11.5%), vaginal discharge (128/664, 19.4%), reduced fetal movement (64/664, 9.7%), uterine contractions (62/664, 9.4%), and suspected leakage of amniotic fluid (54/664, 8.2%). Conversely, 76.4% (507/664) preferred telehealth clinics to in-person visits for prenatal education talks, prenatal and postpartum exercise, and addressing breastfeeding problems. Participants were more comfortable with telehealth clinic tasks for tasks like explaining pregnancy exam results (418/664, 63.1%), self-administering urinary dipsticks at home (373/664, 56.4%), medical history-taking (341/664, 51.5%), and self-monitoring blood pressure using an electronic machine (282/664, 42.8%). %). During the postpartum period, compared to before 18 weeks of gestation, significantly more participants agreed that telehealth clinics could be an option for assessing physical symptoms such as vaginal bleeding (aOR 2.105, 95% CI 1.448-3.059), reduced fetal movement (aOR 1.575, 95% CI 1.058-2.345), uterine contractions (aOR 2.906, 95% CI 1.945-4.342), suspected leakage of amniotic fluid (aOR 2.609, 95% CI 1.721-3.954), fever (aOR 1.526, 95% CI 1.109-2.100), and flu-like symptoms (aOR 1.412, 95% CI 1.030-1.936). They were also more confident with measuring the symphysis-fundal height, arranging further investigations, and making diagnoses with the doctor via the telehealth clinic. The main perceived public health advantage of telehealth clinics was the shorter traveling and waiting time (526/664, 79.2%), while the main concern was legal issues from wrong diagnosis and treatment (511/664, 77.4%).

**Conclusions:**

Face-to-face consultation remained the preferred mode of consultation among the participants. However, telehealth clinics could be an alternative for services that do not require physical examination or contact. An increased acceptance of and confidence in telehealth was found with advancing gestation and after delivery. Enforcing stricter laws and guidelines could facilitate the implementation of telehealth clinics and increase confidence in their use among pregnant women for obstetric care.

## Introduction

Rapidly evolving innovations in technology have enabled efficient and effective communication across the world in numerous ways. This evolution has dramatically revolutionized the delivery of medical care. During the COVID-19 pandemic, the sudden need to reduce disease transmission accelerated the transformation of health care services from traditional in-person consultations to innovative telehealth delivery [1]. Telehealth is defined as the use of digital technology to remotely distribute and facilitate health-related services in an interactive manner [2].

Telehealth has been used in obstetrics to provide prenatal care, maternal and fetal medicine consultations with the interpretation of ultrasound videos or images for fetal anomalies, and genetic counseling [3]. Telemedicine clinics integrated into routine clinical practice could reduce the number of in-person clinical visits, increase patient satisfaction, and decrease prenatal stress without altering other maternal and fetal outcomes [4]. Studies have shown telehealth clinics for prenatal care can lead to higher patient satisfaction [5], improved patient confidence in self-monitoring [6], and cost savings [7]. The potential use of telehealth for providing obstetric care in Hong Kong has not been explored. Therefore, it is important to address patients’ concerns to facilitate its smooth implementation in this field. Accordingly, this questionnaire study aimed to investigate pregnant women’s attitudes, expectations, acceptance, and conception of potential limitations related to the implementation of telehealth obstetric clinical services in Hong Kong.

## Methods

### Overview

This was a prospective cross-sectional questionnaire study carried out at Queen Mary Hospital, Hong Kong. The hospital provides in-person tertiary specialist care for general obstetric and gynecological issues, as well as high-risk pregnancies. It does not currently offer any telehealth obstetric clinics. Regarding standard prenatal care, all pregnant women receive routine prenatal blood tests, Down syndrome screening, a midtrimester morphology scan, a 75-gram oral glucose tolerance test, and group B streptococcus screening with advancing gestations. Women attend the prenatal clinics at regular intervals, where maternal and fetal well-being is assessed through activities such as blood pressure measurement, urine dipstick tests for protein and glucose, measurement of symphysis-fundal height, and checking the fetal heartbeat using a handheld Doppler machine.

In this study, pregnant and postpartum women were identified by research assistants in the prenatal clinic and postpartum wards, respectively. Eligible participants were aged 18 years or older in their first pregnancy or after their first delivery. Multiparous women were excluded from recruitment due to their prior experience with prenatal care, which could potentially lead to differing perceptions and expectations of telehealth in prenatal care. The questionnaire, available in both English and Chinese, was designed by the study team. The participants were required to provide informed consent before answering the questionnaire. They were given the option to complete the questionnaire in either physical or digital format, based on their preference. The questionnaire was anonymous, and pretesting was conducted with pregnant women to ensure that the questions were easily comprehensible. The questionnaire was provided to the participants at various stages of pregnancy: before 18 gestational weeks during their initial prenatal visit, between 24 and 31 gestational weeks during the standard 75-gram oral glucose tolerance test, and after delivery.

### Ethics Approval

This study received ethical approval from the Institutional Review Board of the University of Hong Kong/Hospital Authority Hong Kong West Cluster (UW21-418). All enrolled participants gave informed consent. The study data were anonymous.

### Statistical Analysis

We collected basic demographics including maternal age, education level, ethnicity, socioeconomic status, previous experience with and frequency of using any format of telecommunication, and experiences and frequency of attending telehealth clinics. We investigated participants’ preferences for telehealth clinic services, expectations regarding the implementation of telehealth clinics, perceptions of the feasibility and limitations associated with adopting telehealth clinics, and their privacy concerns. The 5-point rating scale was used accordingly for the required sections. To allow for a 4.5% margin of error at a 95% CI, a sample size of 500 was required. We aimed to enroll roughly equal proportions of participants in the predetermined gestational groups. Statistical analysis was performed using SPSS Statistics software (version 26.0; IBM Corp). Demographic characteristics and questionnaire responses were presented as mean and SD or n and percentage (%). A comparison of questionnaire responses between prenatal and postpartum participants was performed using the chi-square test. Ordinal logistic regression analyses were performed to investigate the association between gestational age and attitudes toward telehealth clinics for outcomes on a 5-point rating scale. Multinomial logistic regression analyses were conducted for categorical outcomes. Covariates (education level, monthly household income, and previous experience with videoconferencing apps) were included in the model for adjusted odds ratios (aORs). *P*<.05 was considered statistically significant.

## Results

### Participant Information

Between November 6, 2021, and August 12, 2022, a total of 670 women were recruited from the clinic and completed the questionnaire. Among them, 6 women were excluded due to having more than 50% of their data missing, leaving 664 participants for the final analysis: 269 (40.5%) before 18 weeks of gestation, 198 (29.8%) between 24 and 31 weeks, and 197 (29.7%) after delivery. Multimedia Appendix 1 contains the participants’ basic demographic information. The majority of the participants were Chinese (622/664, 93.7%), between 31 and 40 years old (476/664, 71.7%), and educated to a tertiary level or above (535/664, 80.6%). Moreover, 93.1% (618/664) had previous experience with videoconferencing apps, and 74.7% (496/664) had heard of telehealth clinics before the questionnaire. However, only 9% (60/664) had attended a telehealth clinic before.

### Preferences Regarding Telehealth Clinic Services

Almost half (329/664, 49.8%) of the participants thought face-to-face consultations were better than telehealth clinics, and only 7.3% (48/664) agreed vice-versa (Table 1). The participants did not show a preference for telehealth clinics over in-person consultations for routine prenatal checkups and certain clinical issues, including vaginal bleeding, vaginal discharge, skin itchiness, reduced fetal movement, uterine contractions, suspected leakage of amniotic fluid, and fever. As gestation advanced (from before 18 weeks to between 24 and 31 weeks) and after delivery, an increasing number of participants began to consider telehealth clinics as a viable option for assessing the aforementioned conditions, although the overall acceptance was still low, at 12.2% (81/664) for routine prenatal checkups, 11.5% (76/664) for vaginal bleeding, 19.4% (128/664) for vaginal discharge, 9.7% (64/664) for reduced fetal movement, 9.4% (62/664) for uterine contractions, and 8.2% (54/664) for suspected leakage of amniotic fluid. In contrast, telehealth clinics were deemed to be an acceptable alternative for prenatal educational talks, prenatal and postpartum exercise classes, and addressing breastfeeding issues when compared to traditional face-to-face visits.

The perceived benefits of telehealth clinics included shorter traveling and waiting times, the possibility of overseas consultations, reduced risk of contracting infectious diseases, improved accessibility, and lower costs. However, the most prevalent concern regarding telehealth clinics was the potential for errors or delays in diagnosis (488/664, 73.8%), followed by challenges with obtaining medication prescriptions (430/664, 65.2%), limited interactive engagement with medical professionals (392/664, 59.3%), privacy issues linked to network security (390/664, 58.8%), discomfort related to exposing private body parts (369/664, 55.7%), and technical problems (304/664, 45.9%).

**Table 1:** Participants’ preferences regarding telehealth clinics^a^.

Questions			Overall (N=664), n (%)	Prenatal <18 weeks (n=269), n (%)	Prenatal between 24 and 31 weeks (n=198), n (%)	Postpartum (n=197), n (%)	Prenatal combined vs postpartum (*P* value)
**In general, do you think telehealth clinics are better than face-to-face clinics?**							.82
	Telehealth clinic is better		48 (7.3)	17 (6.3)	15 (7.6)	16 (8.2)	
	Face-to-face clinic is better		329 (49.8)	134 (50)	100 (50.5)	95 (48.7)	
**To what extent do you prefer telehealth clinics over face-to-face clinics for the following situations?**							
	**Routine prenatal checkup**						.36
		Absolutely prefer/prefer	81 (12.2)	36 (13.4)	21 (10.6)	24 (12.3)	
		Not prefer at all/not prefer	479 (72.4)	196 (72.9)	148 (74.7)	135 (69.2)	
	**Prenatal exercise class**						.43
		Absolutely prefer/prefer	338 (50.9)	139 (51.7)	98 (49.5)	101 (51.3)	
		Not prefer at all/not prefer	154 (23.2)	54 (20.1)	60 (30.3)	40 (20.3)	
	**Prenatal educational talk (eg, breastfeeding or gestational diabetes counseling)**						.63
		Absolutely prefer/prefer	507 (76.4)	202 (75.1)	158 (79.8)	147 (74.6)	
		Not prefer at all/not prefer	56 (8.4)	22 (8.2)	18 (9.1)	16 (8.1)	
	**Postpartum exercise class**						.09
		Absolutely prefer/prefer	350 (53.2)	138 (51.9)	101 (51.8)	111 (56.3)	
		Not prefer at all/not prefer	142 (21.6)	57 (21.4)	53 (27.2)	32 (16.2)	
	**Vaginal bleeding**						<.001
		Absolutely prefer/prefer	76 (11.5)	24 (9)	18 (9.1)	34 (17.3)	
		Not prefer at all/not prefer	497 (75.2)	209 (78.3)	163 (82.7)	125 (63.5)	
	**Vaginal discharge**						.58
		Absolutely prefer/prefer	128 (19.4)	51 (19.2)	36 (18.4)	41 (20.8)	
		Not prefer at all/not prefer	375 (56.9)	149 (56)	120 (61.2)	106 (53.8)	
	**Skin itchiness**						.24
		Absolutely prefer/prefer	199 (30.2)	77 (28.9)	56 (28.6)	66 (33.5)	
		Not prefer at all/not prefer	273 (41.4)	122 (45.9)	79 (40.3)	72 (36.5)	
	**Reduced fetal movement**						.02
		Absolutely prefer/prefer	64 (9.7)	17 (6.4)	19 (9.6)	28 (14.2)	
		Not prefer at all/not prefer	514 (77.8)	218 (81.6)	155 (78.7)	141 (71.6)	
	**Uterine contractions**						<.001
		Absolutely prefer/prefer	62 (9.4)	11 (4.1)	16 (8.1)	35 (17.8)	
		Not prefer at all/not prefer	523 (79.1)	231 (86.5)	162 (82.2)	130 (66)	
	**Suspected leakage of amniotic fluid**						<.001
		Absolutely prefer/prefer	54 (8.2)	10 (3.8)	14 (7.2)	30 (15.2)	
		Not prefer at all/not prefer	535 (81.3)	232 (87.2)	165 (84.6)	138 (70.1)	
	**Fever**						.02
		Absolutely prefer/prefer	181 (27.4)	65 (24.3)	48 (24.4)	68 (34.5)	
		Not prefer at all/not prefer	318 (48.1)	133 (49.8)	103 (52.3)	82 (41.6)	
	**Flu-like symptoms**						.14
		Absolutely prefer/prefer	236 (35.7)	93 (34.8)	63 (32)	80 (40.6)	
		Not prefer at all/not prefer	238 (36)	101 (37.8)	76 (38.6)	61 (31)	
	**Breastfeeding issues**						.21
		Absolutely prefer/prefer	326 (49.4)	131 (49.2)	101 (51.3)	94 (47.7)	
		Not prefer at all/not prefer	162 (24.5)	61 (22.9)	44 (22.3)	57 (28.9)	
**Which of the following factors may encourage you to attend a telehealth clinic instead of a face-to-face clinic?**							
	**Lower cost**						.30
		Totally agree/agree	297 (44.9)	113 (42.4)	88 (44.7)	96 (48.7)	
		Totally disagree/disagree	158 (23.9)	74 (27.6)	44 (22.3)	40 (20.3)	
	**Shorter traveling and waiting time**						.42
		Totally agree/agree	525 (79.1)	213 (79.2)	158 (79.8)	154 (78.2)	
		Totally disagree/disagree	39 (5.9)	21 (7.8)	9 (4.5)	9 (4.6)	
	**Higher accessibility as anyone with internet can access it**						.62
		Totally agree/agree	399 (60.4)	159 (59.6)	116 (58.9)	124 (62.9)	
		Totally disagree/disagree	77 (11.6)	33 (12.4)	24 (12.2)	20 (10.2)	
	**Lower risk of infection with physical distancing**						.35
		Totally agree/agree	421 (63.5)	168 (62.7)	128 (64.6)	125 (63.5)	
		Totally disagree/disagree	73 (11)	35 (13.1)	21 (10.6)	17 (8.6)	
	**Overseas consultation with international experts possible**						.84
		Totally agree/agree	444 (66.9)	180 (66.9)	129 (65.2)	135 (68.5)	
		Totally disagree/disagree	64 (9.6)	27 (10)	19 (9.6)	18 (9.1)	
**Which of the following factors may discourage you from attending a telehealth clinic?**							
	**Privacy issues related to exposing private parts during consultation**						.18
		Totally agree/agree	369 (55.7)	148 (55.2)	110 (55.6)	111 (56.6)	
		Totally disagree/disagree	135 (20.4)	64 (23.9)	39 (19.7)	32 (16.3)	
	**Privacy issues related to network safety**						.28
		Totally agree/agree	390 (58.8)	158 (58.7)	111 (56.1)	121 (61.7)	
		Totally disagree/disagree	104 (15.7)	47 (17.5)	33 (16.7)	24 (12.2)	
	**Difficulty getting medication**						.27
		Totally agree/agree	430 (65.2)	185 (68.8)	122 (62.6)	123 (62.8)	
		Totally disagree/disagree	84 (12.7)	36 (13.4)	26 (13.3)	22 (11.2)	
	**Lack of interactive efficiency with medical professionals**						.99
		Totally agree/agree	392 (59.3)	161 (60.1)	115 (58.1)	116 (59.5)	
		Totally disagree/disagree	80 (12.1)	32 (11.9)	25 (12.6)	23 (11.8)	
	**Risk of errors or delay in diagnosis**						.66
		Totally agree/agree	488 (73.8)	202 (75.4)	146 (74.1)	140 (71.4)	
		Totally disagree/disagree	46 (7)	20 (7.5)	11 (5.6)	15 (7.7)	
	**Technical problems (eg, unstable network)**						.98
		Totally agree/agree	304 (45.9)	121 (45)	94 (47.5)	89 (45.4)	
		Totally disagree/disagree	156 (23.5)	64 (23.8)	46 (23.2)	46 (23.5)	

^a^Chi-square test was performed after removing missing data for each factor.

### Expectations Regarding the Implementation of Telehealth Clinics

Only 24.2% (161/664) of the participants agreed that telehealth clinics should be launched for obstetric services (Table 2). The majority (577/664, 88.1%) preferred live video to web-based text and chat (47/664, 7.2%) or audio (31/664, 4.7%) consultations. The expected public health benefits of telehealth clinics were reduced waiting and traveling times; reduced spread of infectious diseases; reduced workload for medical professionals; increased speed of making diagnoses, referrals, and providing treatment; and higher accessibility to medical services.

**Table 2:** Participants’ expectations regarding telehealth clinics^a^.

Questions			Overall (N=664), n (%)		Prenatal <18 weeks (n=269), n (%)		Prenatal between 24 and 31 weeks (n=198), n (%)		Postpartum (n=197), n (%)			Prenatal combined vs postpartum (*P* value)		
**Do you think telehealth clinics should be launched for obstetric services?**														.82
	Totally agree/agree		161 (24.2)		64 (23.8)		52 (26.3)		45 (22.8)					
	Totally disagree/disagree		237 (35.7)		102 (37.9)		65 (32.8)		70 (35.5)					
**Which of the following format would you prefer for telehealth clinics?**														.53
	Live video consultation		577 (88.1)		235 (88)		171 (89.1)		171 (87.2)					
	Live audio consultation		31 (4.7)		13 (4.9)		6 (3.1)		12 (6.1)					
	Web-based text chat (text and images)		47 (7.2)		19 (7.1)		15 (7.8)		13 (6.6)					
**To what extent do you think the implementation of telehealth clinics will bring the following public health benefits?**														
	**Less workload for medical professionals**												.33	
		Totally agree/agree		357 (53.8)		141 (52.4)		116 (58.6)		100 (50.8)				
		Totally disagree/disagree		118 (17.8)		54 (20.1)		31 (15.7)		33 (16.8)				
	**Higher accessibility of medical services**												.18	
		Totally agree/agree		314 (47.3)		123 (45.7)		87 (43.9)		104 (52.8)				
		Totally disagree/disagree		128 (19.3)		57 (21.2)		38 (19.2)		33 (16.8)				
	**Increased speed of diagnoses, referrals, and treatment**												.03	
		Totally agree/agree		330 (49.7)		132 (49.1)		85 (42.9)		113 (57.4)				
		Totally disagree/disagree		127 (19.1)		60 (22.3)		37 (18.7)		30 (15.2)				
	**Decreased waiting and travel times**												.98	
		Totally agree/agree		526 (79.2)		210 (78.1)		161 (81.3)		155 (78.7)				
		Totally disagree/disagree		33 (5)		14 (5.2)		9 (4.5)		10 (5.1)				
	**Reduced spread of infectious disease (eg, COVID-19)**												.64	
		Totally agree/agree		514 (77.4)		208 (77.3)		157 (79.3)		149 (75.6)				
		Totally disagree/disagree		39 (5.9)		17 (6.3)		8 (4)		14 (7.1)				

^a^Chi-square test was performed after removing missing data for each factor.

### Feasibility and Limitations of Telehealth Clinics

The participants’ perceptions regarding the acceptability of transitioning from the conventional prenatal care model to a telehealth clinic are shown in Table 3. They expressed a high level of comfort with several aspects, including discussing investigation results, conducting urinary dipstick tests at home, participating in history-taking, self-measuring blood pressure with electronic devices, and providing personal information for identity verification. However, they were less confident when it came to self-administering rectal and vaginal swabs under supervision, conducting physical examinations, measuring symphysis-fundal height under supervision, arranging further investigations, listening to fetal heartbeats using a hand-held Doppler machine, and making diagnoses.

**Table 3:** Participants’ perceptions about the feasibility and limitations of telehealth clinics^a^.

Questions			Overall (N=664), n (%)	Prenatal <18 weeks (n=269), n (%)	Prenatal between 24 and 31 weeks (n=198), n (%)	Postpartum (n=197), n (%)	Prenatal combined vs postpartum (*P* value)
**To what extent do you score your confidence in performing the following procedures via telehealth clinic?**							
	**Collecting patients’ personal information and verifying identity**						.03
		Very confident/confident	273 (41.2)	118 (43.9)	85 (42.9)	70 (35.9)	
		Totally unconfident/unconfident	145 (21.9)	61 (22.7)	29 (14.6)	55 (28.2)	
	**Medical history-taking**						.003
		Very confident/confident	341 (51.5)	143 (53.2)	117 (59.1)	81 (41.5)	
		Totally unconfident/unconfident	104 (15.7)	44 (16.4)	20 (10.1)	40 (20.5)	
	**Conducting physical examinations**						.77
		Very confident/confident	60 (9.1)	22 (8.2)	18 (9.1)	20 (10.3)	
		Totally unconfident/unconfident	431 (65.2)	178 (66.2)	129 (65.2)	124 (63.9)	
	**Arranging further investigations (eg, X-ray, microbial tests, blood tests)**						.24
		Very confident/confident	131 (19.8)	48 (17.9)	40 (20.3)	43 (22.1)	
		Totally unconfident/unconfident	365 (55.3)	157 (58.6)	110 (55.8)	98 (50.3)	
	**Making and confirming diagnoses with the doctor**						.06
		Very confident/confident	124 (18.8)	50 (18.7)	28 (14.1)	46 (23.6)	
		Totally unconfident/unconfident	272 (41.2)	128 (47.9)	75 (37.9)	69 (35.4)	
	**Measuring blood pressure with an electronic device at home (results will be interpreted by the doctor)**						.55
		Very confident/confident	282 (42.8)	116 (43.4)	86 (43.4)	80 (41.2)	
		Totally unconfident/unconfident	156 (23.7)	70 (26.2)	43 (21.7)	43 (22.2)	
	**Performing urinary dipstick tests (results will be interpreted by the doctor)**						.24
		Very confident/confident	373 (56.4)	153 (57.1)	118 (59.6)	102 (52.3)	
		Totally unconfident/unconfident	104 (15.7)	44 (16.4)	30 (15.2)	30 (15.4)	
	**Measuring the size of the uterus using a measuring tape at home (supervised by the doctor during telehealth clinic consultation)**						.06
		Very confident/confident	123 (18.6)	43 (16)	35 (17.7)	45 (23.1)	
		Totally unconfident/unconfident	390 (58.9)	176 (65.4)	112 (56.6)	102 (52.3)	
	**Listening to the fetal heartbeat using a hand-held Doppler machine at home (supervised by the doctor during telehealth clinic consultation)**						.30
		Very confident/confident	180 (27.2)	65 (24.2)	54 (27.3)	61 (31.3)	
		Totally unconfident/unconfident	293 (44.3)	134 (49.8)	79 (39.9)	80 (41)	
	**Self-administering swabs in the rectum and lower vagina at home (supervised by the doctor during telehealth clinic consultation)**						.12
		Very confident/confident	82 (12.4)	29 (10.8)	21 (10.6)	32 (16.4)	
		Totally unconfident/unconfident	461 (69.6)	198 (73.6)	132 (66.7)	131 (7.2)	
	**Discussing investigation results (eg, Down syndrome screening, oral glucose tolerance test)**						.41
		Very confident/confident	418 (63.1)	176 (65.4)	126 (63.6)	116 (59.5)	
		Totally unconfident/unconfident	107 (16.2)	46 (17.1)	28 (14.1)	33 (16.9)	

^a^Chi-square test was performed after removing missing data for each factor.

### Privacy And Medicolegal Perspectives Regarding Telehealth Clinics

Half (336/664, 50.8%) of the participants believed that the current laws and guidelines were insufficient to protect patients’ rights (Table 4). A significant proportion of the participants were worried about legal issues stemming from wrongful diagnosis and treatment (511/664, 77.4%), inappropriate use of the telehealth consultation records (464/664, 70.1%), and leaking of personal information (401/664, 60.6%). Moreover, 47.1% (312/664) agreed the consultation should be recorded by the patients, and 58.2% (385/664) expected a written record following a telehealth consultation. The majority of participants agreed that new legislation should be established to govern the protection of confidentiality (531/664, 80.2%), acquisition of consent (541/664, 81.7%), and storage of medical records (522/664, 78.9%). More participants demonstrated confidence in the implementation of telehealth clinics when accompanied by the enforcement of stricter laws and guidelines.

**Table 4:** Participants’ perceptions of privacy and medicolegal issues surrounding telehealth clinics^a^.

Questions			Overall (N=664), n (%)	Prenatal <18 weeks (n=269), n (%)	Prenatal between 24 and 31 weeks (n=198), n (%)	Postpartum (n=197), n (%)	Prenatal combined vs postpartum (*P* value)
**Do you think current laws and guidelines are sufficient to protect a patient’s rights when using a telehealth clinic?**							.08
	Totally agree /agree		83 (12.6)	34 (12.6)	21 (10.7)	28 (14.4)	
	Totally disagree /disagree		336 (50.8)	150 (55.8)	100 (50.8)	86 (44.1)	
**When using telehealth clinics, how worried are you about facing the following scenarios?**							
	**Leakage of personal information and verifying patient’s identity**						.11
		Very worried/worried	401 (60.6)	171 (63.6)	112 (56.6)	118 (60.5)	
		Totally not worried/not worried	93 (14)	46 (17.1)	27 (13.6)	20 (10.3)	
	**Inappropriate use of the telehealth consultation record (eg, for marketing)**						.01
		Very worried/worried	464 (70.1)	201 (74.7)	139 (70.2)	124 (63.6)	
		Totally not worried/not worried	72 (10.9)	31 (11.5)	21 (10.6)	20 (10.3)	
	**Legal issues arising from misdiagnosis and wrong treatment provided in the telehealth clinic**						.04
		Very worried/worried	511 (77.4)	213 (79.8)	154 (77.8)	144 (73.8)	
		Totally not worried/not worried	35 (5.3)	16 (6)	12 (6.1)	7 (3.6)	
**Do you think that more new legislation should be introduced on the following issues with the implementation of telehealth clinics?**							
	**Confidentiality of personal data**						<.001
		Totally agree/agree	531 (80.2)	228 (84.8)	164 (82.8)	139 (71.3)	
		Totally disagree/disagree	28 (4.2)	9 (3.3)	9 (4.5)	10 (5.1)	
	**Obtaining consent for recording the telehealth consultation**						<.001
		Totally agree/agree	541 (81.7)	235 (87.4)	164 (82.8)	142 (72.8)	
		Totally disagree/disagree	21 (3.2)	4 (1.5)	8 (4)	9 (4.6)	
	**Storage of videos from the telehealth consultation**						<.001
		Totally agree/agree	522 (78.9)	226 (84)	160 (80.8)	136 (69.7)	
		Totally disagree/disagree	30 (4.5)	10 (3.7)	8 (4)	12 (6.2)	
**Do you think telehealth consultations should be recorded by pregnant women?**							.82
	Totally agree/agree		312 (47.1)	120 (44.6)	102 (51.5)	90 (46.2)	
	Totally disagree/disagree		161 (24.3)	74 (27.5)	41 (20.7)	46 (23.6)	
**Do you think the telehealth consultation should be recorded by the doctors as part of the medical record?**							.30
	Totally agree/agree		232 (35.0)	81 (30.1)	74 (37.4)	77 (39.5)	
	Totally disagree/disagree		227 (34.3)	104 (38.7)	61 (30.8)	62 (31.8)	
**Do you think a written record of the consultation should be provided to the patient?**							.78
	Totally agree/agree		385 (58.2)	147 (54.6)	123 (62.4)	115 (59)	
	Totally disagree/disagree		91 (13.8)	44 (16.4)	23 (11.7)	24 (12.3)	
**Would your confidence in the successful implementation of telehealth clinics be enhanced with the enforcement of stricter laws and guidelines?**							.97
	Totally agree/agree		363 (54.8)	141 (52.4)	114 (57.6)	108 (55.4)	
	Totally disagree/disagree		74 (11.2)	34 (12.6)	19 (9.6)	21 (10.8)	

^a^Chi-square test was performed after removing missing data for each factor.

### Perceptions of and Confidence in Telehealth Clinics Across Gestations and After Delivery

Table 5 demonstrates how participants’ perceptions of and confidence levels regarding telehealth clinics evolved in relation to prenatal issues and procedures as pregnancy progresses and after childbirth. The acceptance of attending telehealth clinics increased with the experience of pregnancy and delivery. During the postpartum period, compared to before 18 weeks of gestation, significantly more participants agreed that telehealth clinics could be an option for assessing physical symptoms such as vaginal bleeding (aOR 2.105, 95% CI 1.448-3.059), reduced fetal movement (aOR 1.575, 95% CI 1.058-2.345), uterine contractions (aOR 2.906, 95% CI 1.945-4.342), suspected leakage of amniotic fluid (aOR 2.609, 95% CI 1.721-3.954), fever (aOR 1.526, 95% CI 1.109-2.100), and flu-like symptoms (aOR 1.412, 95% CI 1.030-1.936). In addition, their confidence levels grew when it came to tasks like measuring the symphysis-fundal height, arranging further investigations, and making diagnoses with the doctor via the telehealth clinic. However, they exhibited lower confidence levels when it came to medical history-taking.

**Table 5:** Comparison between participants’ prenatal and postpartum status and attitudes toward telehealth clinics.

Items			*P* value		Unadjusted OR^a^ (95% CI)		*P* value		Adjusted OR (95% CI)^b^
**Prefer telehealth clinic over face-to-face clinic**									
	Vaginal bleeding	<.001		2.288 (1.592-3.289)		<.001		2.105 (1.448-3.059)	
	Reduced fetal movement	.008		1.670 (1.140-2.445)		.03		1.575 (1.058-2.345)	
	Uterine contractions	<.001		2.933 (1.997-4.309)		<.001		2.906 (1.945-4.342)	
	Suspected leakage of amniotic fluid	<.001		2.718 (1.821-4.056)		<.001		2.609 (1.721-3.954)	
	Fever	.008		1.525 (1.116-2.084)		.009		1.526 (1.109-2.100)	
	Flu-like symptoms	.049		1.361 (1.001-1.852)		.03		1.412 (1.030-1.936)	
**Feel confident performing the following procedures via telehealth clinic**									
	Medical history-taking	<.001		0.579 (0.423-0.794)		.01		0.634 (0.459-0.876)	
	Arranging further investigations (eg, X-ray, microbial tests, blood tests)	.11		1.298 (0.944-1.785)		.048		1.390 (1.004-1.924)	
	Making and confirming diagnoses with the doctor	.02		1.456 (1.065-1.991)		.02		1.478 (1.075-2.033)	
	Measuring the size of the uterus using a measuring tape by pregnant women at home (supervised by the doctor during telehealth clinic consultation)	.02		1.475 (1.068-2.038)		.01		1.529 (1.100-2.126)	

^a^OR: odds ratio.

^b^Adjusted for education level, monthly household income, and previous experience with video conferencing apps.

## Discussion

### Principal Results

In this study, we found that pregnant women preferred a face-to-face visit (329/664, 49.8%) to a telehealth consultation (48/664, 7.3%). Telehealth clinics were less favored by the participants for physical complaints and routine prenatal care, but they were deemed a desirable option for prenatal educational talks, disclosure of investigation results, postpartum exercise classes, and discussing breastfeeding problems. With advancing gestation and after delivery, we found an increasing acceptance of and confidence in telehealth for tackling certain pregnancy-related physical symptoms and prenatal procedures. The perceived advantages of telehealth clinics were saving time for waiting and transportation, making international consultations feasible, and reducing exposure to infectious diseases. However, the risk of making a wrong diagnosis, lack of interactive efficiency, barriers to obtaining medication prescriptions, exposure of private parts, and wrongful disclosure or misuse of personal information were the main concerns regarding telehealth clinics. Only 24.2% (161/664) of the participants agreed that telehealth clinics should be launched for obstetric services. Participants also felt that enforcing stricter laws and guidelines could facilitate the implementation of telehealth clinics and increase confidence in its use among pregnant women.

### Interpretation and Implications

Modern technology enables the prompt distribution of information. There is an expectation that cutting-edge technology can be incorporated into conventional health care to assist in patient care and reduce the need for unnecessary in-person visits. During the COVID-19 pandemic, the imperative to maintain public health without interrupting essential services prompted the exploration and implementation of telehealth. In this context, we examined the willingness to transition traditional in-person prenatal care to telehealth clinics, gauging participants’ confidence in conducting routine prenatal procedures at home. Automated devices designed for self-monitoring of blood pressure have been validated for use during pregnancy [8]. While women can measure symphysis-fundal height themselves, it is worth noting that this method may yield higher intraobserver variations and readings compared to measurements taken by midwives [9]. The feasibility of fetal heart monitoring with a Doppler machine at home has been explored, particularly for identifying fetuses at risk of congenital heart block in pregnant women with anti-Ro antibodies [10,11]. Self-sampling for group B streptococcus screening has also proven to be achievable [12]. A prenatal care model was proposed for restructuring prenatal visits while still delivering essential services. Physical visits were essential for specific appointments, including early pregnancy examinations, fetal viability and dating, a morphology scan at 19 weeks, screening for gestational diabetes and anemia at 28 weeks, pertussis vaccination, group B streptococcus screening and confirming fetal presentation at 36 weeks, conducting cervical assessments, and discussing postterm management at 39 weeks. In between these critical in-person visits, a total of 5 telehealth visits were conducted to monitor maternal vitals and fetal heart rate, as well as address any questions [1]. A randomized controlled trial conducted at a single center found that incorporating telehealth visits alongside fewer physical consultations (8 physical visits and 6 telehealth visits) led to higher patient satisfaction and lower prenatal stress compared to standard conventional prenatal care (12 physical visits). Notably, this shift did not compromise maternal and neonatal outcomes [4].

Another large-scale study examined the application of telehealth in obstetric services in Australia, comparing conventional physical visits to integrated visits. Among women receiving integrated care, only 3 physical visits were needed for low-risk cases and 5 for high-risk cases. Approximately 50% of the consultations were replaced by telehealth visits, and there was no significant difference in the incidence of fetal growth restriction, preeclampsia, gestational diabetes, and stillbirth between both groups [13].

Nulliparous women in their first and early stages of pregnancy often experience anxiety and lack confidence in self-monitoring, examinations, and managing minor pregnancy concerns. We aimed to understand how different pregnancy experiences influence perceptions of telehealth clinics. As gestation progressed and after delivery, the participants became more familiar with prenatal visit procedures and grew more confident in handling their pregnancies. Thus, they were more open to the idea of telehealth consultations as an option for prenatal monitoring and addressing minor concerns, such as measuring symphysis-fundal height, addressing vaginal bleeding, or monitoring reduced fetal movement. Overall, participants who experienced telehealth clinics reported high satisfaction [5,14]. The acceptance of various prenatal programs varied among the participants, and they were generally more comfortable with telecommunication for services that did not require physical examinations or direct contact, such as educational talks, exercise classes, and addressing breastfeeding concerns. It is important to acknowledge that telehealth clinics cannot completely replace conventional prenatal care, and flexibility should be maintained for women who desire face-to-face consultations.

Understanding patients’ concerns is crucial to customizing care and ensuring the successful implementation of telehealth. Common barriers and anxieties revolve around doctor-patient relationships, the inability to conduct physical examinations, the quality of care, patient confidentiality, cybersecurity, legal and regulatory considerations, and the roles of both clinicians and patients [15]. To address these concerns, there are international and local ethical guidelines available to guide service providers and protect patients’ rights [16,17]. In short, the same principles and standards of care that protect patients during in-person consultations equally apply to telemedicine. Telemedicine should be conducted through a structured and organized platform by adequately trained doctors. Obtaining informed consent is essential to outline the objectives and limitations of telemedicine, as well as to address issues of confidentiality and data protection. Consultation should include appropriate evaluation and treatment, with proper documentation. Moreover, efforts should be made to maintain the doctor-patient relationship to the greatest extent possible.

### Strengths and Limitations

The major strength of this study lies in its comparison between women in the antepartum and postpartum periods, shedding light on potential changes in perception and confidence over time. However, this study has several limitations to consider.

First, this study may not have captured the views of the views of ethnic minoritized groups, as the data collection was conducted in Chinese and English, potentially excluding those who do not read those languages. In addition, approximately 80% (535/664) of the participants had a higher level of education, which could affect the generalizability of our findings.

Second, the perspectives of multiparous women, who have prior pregnancy and birthing experiences, might differ from those of nulliparous women. However, we decided to solely evaluate nulliparous women to minimize heterogeneity. Additionally, the postpartum group in our study could provide some insights into the views of multiparous women.

Third, this study did not explore the potential influence of pregnancy complications on participants’ perceptions of telehealth. Women who experience such pregnancy complications might prefer traditional face-to-face consultations. Finally, the participants did not actually experience an obstetric telehealth clinic, which could have positively influenced their perception and acceptance afterward. Our next step could involve an evaluation study to gather their views and satisfaction after attending a telehealth clinic.

Obstetric care is unique because it requires frequent and close monitoring during pregnancy to detect asymptomatic disease early and improve pregnancy outcomes. For example, monitoring blood pressure and urine protein levels helps screen for preeclampsia, and monitoring blood glucose levels is essential for women with gestational diabetes. However, factors such as COVID-19 vaccine hesitancy, concerns about COVID-19–related complications, and uncertainties about the long-term impact of vaccines or infections on the fetus may lead pregnant individuals to isolate themselves and avoid seeking medical help [18]. In such situations, telehealth could be an ideal model to facilitate prenatal care, ensuring that women can access necessary care even when they are hesitant to seek in-person care.

### Conclusion

While face-to-face consultations remained the preferred mode of care, telehealth clinics could be an alternative for services that do not require physical examination or contact. Notably, acceptance of and confidence in telehealth grew as pregnancy progressed and after delivery.

To prepare for future unforeseeable infection outbreaks, there is a pressing urgency to incorporate telehealth in the obstetric care model. Various stakeholders and government bodies should invest in and develop a comprehensive telehealth system as a supplementary health care service. Enforcing stricter laws and guidelines can facilitate the implementation of telehealth clinics and increase pregnant women’s confidence in using them for obstetric care. This is a crucial step in laying the groundwork for addressing potential disruptions to face-to-face services and ensuring continuity of care provision should isolation and social distancing measures become necessary in the future.

## Appendix

Multimedia Appendix 1 Basic demographics of the survey respondents (N=664).

## Abbreviations

aOR: adjusted odds ratio

## Data Availability

The data sets generated during and/or analyzed during this study are available from the corresponding author upon reasonable request.
