# Supplementary material for: Perceptions and Challenges of Telehealth Obstetric Clinics Among Pregnant Women in Hong Kong: Cross-Sectional Questionnaire Study
Source: J Med Internet Res. 2023 Sep 19;25:e46663. doi: 10.2196/46663 (PMC10548321; doi:10.2196/46663)
Supplement: Multimedia Appendix 1 [file jmir_v25i1e46663_app1.docx]

Supplementary Table. Basic demographics of all survey respondents (n=664)

|  | n (%) |
| --- | --- |
| Maternal age |  |
| - < 20 | 3 (0.5%) |
| - 20-30 | 166 (25.0%) |
| - 31-40 | 476 (71.7%) |
| - 41-50 | 19 (2.9%) |
| Ethnicity |  |
| - Chinese | 622 (93.7%) |
| - Asian other than Chinese | 27 (4.1%) |
| - Caucasian | 13 (2.0%) |
| - Others | 2 (0.3%) |
| Education level |  |
| - Below primary | 1 (0.2%) |
| - Primary | 2 (0.3%) |
| - Secondary | 126 (19.0%) |
| - Tertiary or above | 535 (80.6%) |
| Monthly household Income |  |
| - Below HK$20,000 | 61 (9.2%) |
| - HK$20,000-$50,000 | 243 (36.6%) |
| - HK$50,000-$100,000 | 227 (34.2%) |
| - HK$100,000-$200,000 | 103 (15.5%) |
| - Above HK$200,000 | 24 (3.6%) |
| - Missing | 6 (0.9%) |
| Are you a medical professional? |  |
| - Yes | 36 (5.4%) |
| - - Nurse | 24 (3.6%) |
| - - Patient Care Assistant | 6 (0.9%) |
| - - Others | 6 (0.9%) |
| - No | 625 (94.1%) |
| - Missing | 3 (0.5%) |
| Antenatal < 18 weeks (n=269) |  |
| - Gestational Age (weeks), median (IQR) | 14.57 (14.14 -15.14) |
| Antenatal 24-31 weeks (n=198) |  |
| - Gestational Age (weeks), median (IQR) | 26.86 (26.43-27.43) |
| Postnatal (n=197) |  |
| - Days after delivery (days), median (IQR) | 1 (1-2) |
| Have you ever used any video conferencing applications before (Zoom, Google Meet, Skype etc.)? |  |
| - Yes | 618 (93.1%) |
| - No | 46 (6.9%) |
| How often do you used video conferencing applications (per month)? |  |
| - Less than 5 times | 397 (59.8%) |
| - 5-10 times | 145 (21.8%) |
| - More than 10 times | 122 (18.4%) |
| Have you heard of virtual clinics? |  |
| - Yes | 496 (74.7%) |
| - No | 166 (25.0%) |
| - Missing | 2 (0.3%) |
| Where did you learn about virtual clinics? (Multiple choices) |  |
| - News | 156 (23.5%) |
| - Relatives or friends | 89 (13.4%) |
| - Books or magazines | 13 (2.0%) |
| - Hospitals or clinics | 152 (22.9%) |
| - TV programs | 60 (9.0%) |
| - Internet | 217 (32.7%) |
| - Others | 17 (2.6%) |
| Do you think virtual clinics are common in Hong Kong? |  |
| - Very common / Common | 125 (18.8%) |
| - Neutral | 506 (76.2%) |
| - Totally uncommon / Uncommon | 26 (3.9%) |
| - Missing | 7 (1.1%) |
| Have you ever tried virtual clinic services before? |  |
| - Yes | 60 (9.0%) |
| - No | 604 (91.0%) |
| How many times have you ever used virtual clinic services? |  |
| - Once | 31 (4.7%) |
| - Twice | 18 (2.7%) |
| - Three times | 5 (0.8%) |
| - Four times | 0 (0.0%) |
| - > four times | 6 (0.9%) |
